# Attentional influences on cue weighting in vowel perception: Examining prosodic prominence and informational masking

**DOI:** 10.3758/s13414-025-03123-5

**Published:** 2025-07-22

**Authors:** Wei Zhang, Jeremy Steffman

**Affiliations:** 1 School of Foreign Studies, Nanjing University, Nanjing, China; 2 Department of Linguistics, McGill University, Montreal, Canada; 3 School of Philosophy, Psychology and Language Sciences, The University of Edinburgh, 3 Charles St, EH8 9AD Edinburgh, UK

**Keywords:** Speech perception, Cue weighting, Attention, Vowel perception, Prominence

## Abstract

Beyond sources of listener-external variability such as variation in talker and acoustic context, listener-internal variation also plays a role in speech perception and cue weighting. The present study examines the effects of prosodic prominence, signaled by F0, and multi-talker babble noise as methods of boosting and decrementing listeners’ attention, respectively. Listeners categorized four English vowel contrasts, including two high vowel contrasts and two non-high vowel contrasts, with both formant cues and vowel duration varying along a continuum. In Experiment 1, results showed that prominence boosted formant cue usage, whereas babble noise was detrimental to formant cue usage, aligning with predicted roles in modulating listener attention. Listeners’ use of vowel duration, a secondary cue to the contrasts, was also impacted by prominence or babble noise. In Experiment 2, two methods of eliciting F0-based prominence, off-target (contextual) and on-target (target-internal), were investigated. Results showed that off-target prominence showed a very limited effect in boosting formant cue usage. Results are discussed in terms of the role of prosodic prominence in speech perception, and the role of attention in perceptual processing. The data and code for the experiments is available on the OSF at:https://osf.io/52khc/.

## Introduction

Phonological contrasts are distinguished by multiple acoustic dimensions in speech (e.g., Lisker, 1986). One benefit of such multi-dimensional and redundant distinctions is to accommodate variations in speech communication. Beyond sources of listener-external variability, such as talker- and context-specific realization of phonological contrasts, listener-internal variation also plays a role, mediating the process of mapping acoustic information in the speech input to linguistic representations.

One relatively well-studied source of listener-internal variation is in the listener’s attentional resources (e.g., Gordon et al., 1993). For example, speech perception may become less accurate, and more reliant on contextual information when distractors, whether auditory, visual, or otherwise, are present (Gordon et al., 1993; Mattys & Wiget, 2011; Mattys et al., 2013b). A decrease in attentional resources, or a relative increase in cognitive load during speech perception, has been argued to be detrimental to sub-lexical phonetic processing (Mattys et al., 2013a), with documented negative consequences as measured with word segmentation and phoneme monitoring tasks (Fernandes et al., 2010; Wurm & Samuel, 1997). Interpretation of the speech signal thus appears to depend on the amount of attention the listener gives to the target speech, and furthermore, the particular dimension in the speech signal that the listener pays more attention to (Gordon et al., 1993).

The present study examines how two factors affecting the allocation of listeners’ attention impact the perception of American English vowel contrasts, with a focus on the relative usage of primary and secondary cues and their relation to vowel height, which has previously been shown to mediate cue weighting (Steffman & Zhang, 2023). The first factor is prosodic prominence, which has been said to attract listeners’ attention to the prosodically prominent region of the speech signal (De Jong, 2004; Cole & Jakimik, 1980a, b; Steffman & Zhang, 2023). The second factor is (babble) noise, which distracts listeners’ attention from the target vowel (Mattys et al., 2013b). Note that in this study we use the term “attention” to reference an abstract cognitive process that reflects how listeners allocate their focus on acoustic cues during speech perception tasks, without reference to specific neurobiological mechanisms. In that sense, our definition is not mechanistic, and we do not focus our study on mechanisms that may underlie the phenomena that we refer to abstractly as resulting from differences in attention. In examining these two influences and their interaction, we pursue a fuller understanding of how speech perception in noise, and in the context of prosodic prominence, operates, and how both of the factors mediate cue usage.

Prosodic prominence is one of the means by which speakers can emphasize regions in the speech signal. Cross-linguistically, and for different categories of speech sounds, prosodic prominence manifests in various ways. Most relevant to the present study, for (American English) vowels, prosodic prominence can be realized by more extreme movements of the articulators, and by higher F0 and longer duration (e.g., Beckman & Edwards, 1994; Cho, 2005; De Jong, 2004; Erickson, 2002; Kim & Arnhold, 2024). These modulations are indeed taken into account by listeners when categorizing prominent vowels, described in Section “Prominence and its impact on speech perception”. Given the relative nature of prominence perception (Cole et al., 2010), decreasing the salience of the context can, in principle, increase the salience of the target, and prominence in both speech production and speech perception has been suggested to be relational in this sense (Cangemi & Baumann, 2020; Cole et al., 2019; Roessig, 2023). In this vein, other methods of directing attention through relative prominence, such as manipulating the salience of context, have also been examined to some extent (e.g., Steffman, 2020; 2021b). However, combined testing of context and target-based prominence marking, and their interaction, has not been carried out to our knowledge. This study thus aims to extend the understanding of prominence effects on vowel perception by examining the contribution of both vowel-internal and contextual prominence, and their interaction with variation in distractors in the speech signal.

Contrary to prosodic prominence, distractors can direct listeners’ attention away from the target speech, and effectively mediate the way the speech signal is perceived. For example, Gordon et al. (1993) compared cue usage in categorization between an ideal listening condition (i.e., high attention condition) and a distractor condition (i.e., low attention condition), testing perception of a vowel contrast and a stop contrast in English. Distractors were introduced by asking the participant to solve math problems or perform visual judgment tasks concurrently with the identification task. The contribution of the primary cue (VOT for stops, formants for vowels) was reduced in the presence of distractors, whereas that of a secondary cue (post-stop F0 for stops, vowel duration for vowels) was boosted in the distractor condition relative to the ideal condition. As detailed in the Section “Speech categorization in adverse listening conditions” below, results of subsequent studies have been mixed regarding whether the reliance on primary versus secondary cues is enhanced, reduced, or unaffected by distractors. The present study therefore aims to further investigate the relative utilization of cues in vowel contrast categorization under conditions of low attention introduced by co-occurring multi-talker babble noise. In doing so, we also consider possible mitigating effects of prosodic prominence, which are predicted to draw attentional resources to the target vowels.

In what follows, we overview the concept of prominence and its effects on speech in Section “Prominence and its impact on speech perception” and the influence of noise in speech perception will be addressed in Section “Speech categorization in adverse listening conditions”.

### Prominence and its impact on speech perception

The function of prosodic prominence in the perception of speech segments has been examined in a variety of previous studies. First, in line with the understanding of prosodic prominence as orienting listener attention to particular segments (De Jong, 2004), various studies have shown benefits in recognition under prominence (e.g., Bond & Garnes 1980; Cole & Jakimil, 1980b; Mehta & Cutler, 1988). These benefits, induced by both context-based and target-internal prominence cues, include more accurate identification for segments (Cole & Jakimik, 1980a) and more rapid identification measured in phoneme monitoring, gating tasks, and eyetracking (Cutler, 1976; Akker & Cutler, 2003; Steffman, 2023). Beyond these benefits in recognition accuracy and speed, prominence has also been examined in light of the way that it systematically structures segmental realization in speech, as in more extreme formant patterns for vowels when they are produced as prominent (Cho, 2005; Erickson, 2002; Foster & Cole, 2024). Reflecting these production patterns, listeners have been shown to adjust their categorization of vowels such that perceptual boundaries between two vowels become more peripheral in the vowel space when prominent (Steffman, 2021b, 2023). These effects can be considered compensatory in the sense that listeners are compensating for the effects of prominence on vowel acoustics, and recalibrating their perception of vowel contrasts accordingly.

Most relevant to the present study, Steffman and Zhang (2023) documented both attention-orienting and compensation phenomena caused by prominence, as elicited by pitch accent variation on the target vowel, which we call *on-target* modulation of prominence. That study tested categorization of four vowel contrasts while varying formant cues, which are typically considered the primary cue to vowel contrasts, as well as duration, which serves as both a secondary cue to the vowel contrasts (Hillenbrand et al., 2000) and an indicator of prominence (Fry, 1958). The predicted directionality of the duration and f0 effects on the categorization of the high and non-high vowel contrasts was outlined (Steffman & Zhang, 2023, Table 1). Steffman and Zhang (2023) found that high pitch accent and longer duration, both of which are indicators for prominence, enhanced the utilization of formant cues in vowel categorization. This aligns with the claimed function of prominence for listeners (Cole & Jakimik, 1980a; De Jong, 2004), raising the perceptual salience of the vowel and directing more of the listener’s attention to the primary cue. In Steffman and Zhang (2023), listeners also adjusted their perception of the vowel contrasts, mirroring acoustic peripheralization of vowels in the vowel space, further indicating that they are highly sensitive to prominence-related modulation of vowels in their acoustic realization.

As prominence is a relative feature, in which speech is perceived relative to its context, the same compensatory shifts in vowel categorization have been elicited on the basis of contextual variation in prominence, which we call *off-target* modulation of prominence (Steffman, 2021a, b). Preceding context has also been shown to be required for facilitatory effects of prominence in phoneme monitoring (Rysling et al., 2020), underscoring that off-target contextual prominence cues have an impact in speech perception. Moreover, while both on-target and off-target effects of prominence have been shown for the compensatory effects mentioned above, the interaction of on-target and off-target prominence has not been explored. Cue use enhancement, as tested for on-target prominence in Steffman and Zhang (2023), has not been tested for contextual/off-target prominence. The present study thus examines the interaction between on-target and off-target prominence, and possible enhancement effects of both, extending our understanding of the role of prosodic prominence in speech perception.

### Speech categorization in adverse listening conditions

As noted above, cue weighting in speech perception is influenced by the listener’s attentional state. Recall that Gordon et al. (1993) found that in a math problem-solving dual-task condition, primary cue usage was reduced while secondary cue usage was boosted compared to an ideal listening condition. Kong and Lee (2018) observed that in a dual-task condition, cue weighting of the primary cue, Voice Onset Time (VOT), in the categorization of Korean laryngeal stops was hindered, whereas that of the secondary cue, Fundamental Frequency (F0), was not affected compared to the ideal listening condition. In another study, Kim (2019) investigated the categorization of /$\upepsilon$/-/æ/ in English, both with and without a concurrent visual search task. Unlike Gordon et al. (1993) and Kong and Lee (2018), Kim (2019) found that in the dual-task condition, both the primary cue (formant) and the secondary cue (duration) were *boosted* compared to the ideal listening condition. Findings from these three studies suggest that in categorization tasks where listeners’ attention is divided by a concurrent dual task, it is globally less clear how their reliance on primary and non-primary cues changes compared to the ideal listening condition. Furthermore, as highlighted by Symons et al. (2023), distractors involving dual tasks such as math problem-solving are less common in real-world listening scenarios than those involving noise or multi-talker situations. In their study, Symons et al. (2023) thus examined the effect of multi-talker babble noise on cue weighting in the categorization of a stop contrast in English. They found that babble noise increased secondary cue usage but showed no effect on primary cue reliance.

The current study further examines vowel perception in non-ideal listening conditions. Following Symons et al. (2023), we employ multi-taker babble noise to induce a low-attention listening condition. Multi-talker babble noise can interfere with the perception of the target sound as it contains audible linguistic and semantic information. Thus, it can be seen as a way of ‘informational masking’, where the distracting sound makes it harder to direct full attention to the target sound (Freyman et al., 1998). Another type of noise is ‘energetic masking’, such as white noise or speech-shaped noise (Chen et al., 2023), which lacks linguistic or semantic information but interferes with the perception by degrading the target signal (Arbogast et al., 2002). Speech-shaped noise can also affect perceptual cue weighting in speech categorization (Mattys, 2004; Winn et al., 2013; Varnet et al., 2016; Wu & Holt, 2022; DiNino, 2024). However, because this study focuses on noise’s higher-level effect on attention, we opted to use multi-talker babble noise. We predicted that perceptual cue weights in vowel categorization will shift between the multi-talker babble noise condition and an ideal listening condition without noise. Previous dual-task experiments and the only babble noise experiment on perceptual cue weighting (to our knowledge) yield mixed results regarding the reduction or boosting of cue usage for either primary or secondary cues.

However, based on previous findings that prosodic prominence, which can be considered to raise attention, boosts cue usage for formants (Steffman & Zhang, 2023), we predict that babble noise will, conversely, reduce the cue usage of formants. In other words, we predicted that these effects operate in opposition to each other, based on their oppositional influences on listener attention. While prominence did not boost duration cue usage in Steffman and Zhang (2023), it remains an open question if babble noise will impact this cue.

### The current study

We tested categorization of four English vowel contrasts (two high vowel contrasts and two non-high vowel contrasts), focusing on listeners’ relative reliance on the primary cue, formants, and non-primary cue, duration, and their interaction with vowel height. Two experiments are conducted in this study. The first one examines the effects of both noise and prominence on formant and durational cue perception. In the second, the impact of on-target versus off-target prominence modulation and their interactions are examined to better understand the role of contextual prominence in attention modulation. The design is described below in Section “Methods”.

## Methods

In this section, we detail the experimental methods for both experiments presented in this paper, as they are largely similar. In both, we presented stimuli to listeners, which they categorized as one of two English words in a two-alternative forced-choice identification task. Following Steffman and Zhang (2023), we tested four vowel contrasts, exemplified with eight target words, all in the consonant frame /k_d/. These were “keyed” /i/ vs. “kid” /ɪ/, “cooed” /u/ vs. “could” /$\mho$/, “ked” (a type of insect) /$\upepsilon$/ vs. “cad” /æ/, and “cud” /$\wedge$/ vs. “cod” /$\text {a}$/.

### Materials

#### Experiment 1

The stimuli for both studies were adapted from those used in Steffman and Zhang (2023), and were created via formant resynthesis. The resynthesis varied F1/F2 (jointly varied), F0, and duration. F1/F2 resynthesis was based on the speaker’s production of each vowel. The speaker who produced the stimuli was a female speaker of American English, born and raised in the eastern US. The speaker was recorded in a sound-attenuated room with a Shure SM27 large-diaphragm condenser microphone and pop filter at 44.1 kHz and 32-bit depth. The speaker produced the phrase “I’ll say *x* now” (*x* being one of the eight target words) with four different levels of emphasis on the target word *x* (no-emphasis, emphasized, more emphasized, very emphasized). The emphasis manipulation was intended to elicit different levels of F0-based prominence, which were used in resynthesis of the F0 conditions.

**Fig. 1:**
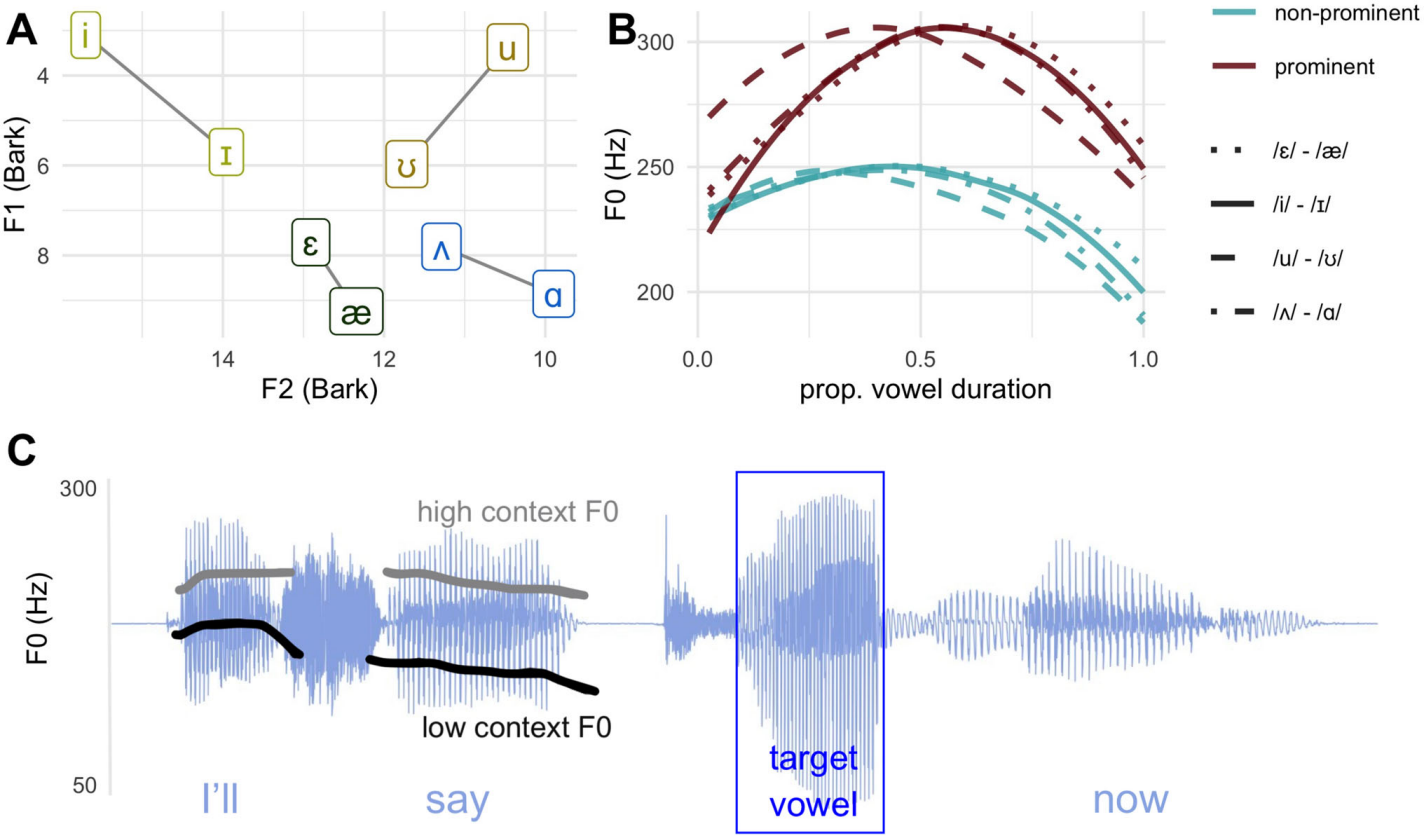
**A** Formant continuum endpoints, in F1$\times$F2 Bark space for each of the four vowel contrasts, which are differentiated by coloration and connected by lines. **B** F0 contours for each contrast (line type) and prominence condition (line color), over the normalized duration of the target vowels. **C** The carrier phrase from the stimuli in Experiment 2, shown as a waveform with overlaid F0 traces on the pre-target material (F0 range indicated at left). The displayed waveform is for the low context precursor, and the word “kid” (/ɪ/ endpoint of the high front vowel contrast continuum). Note that the F0 ranges are different in panels B and C, and that the word “now” was the same in all conditions

There were originally seven steps in each vowel quality continuum. F1 and F2 were manipulated by LPC-based decomposition and resynthesis with the Burg method and using a Praat script (Winn, 2019), and the following seven-step continua were generated: “kid-keyed,” “ked-cad,” “could-cooed,” and “cud-cod.” Vowels in “kid,” “ked,” “could,” and “cud” were the base for resynthesis, using these produced F1/F2 values as end points and interpolating to the F1 and F2 for the other member in the pair. All manipulations occurred within the vowel segment of the base file for a given pair, and the consonants of the target words remained constant within a pair. The F1 and F2 of the continuum end points for each contrast are displayed in Fig. 1A.

**Table 1:** Stimuli values for formants and F0 shown for the continuum endpoints, labeled as vowel at left. Measurements for formants are shown in both Hz and Bark, and F0 measures are given as the mean and maximum f0 values, in Hz, for both prominent and non-prominent conditions. Note that each vowel pair in a contrast had the same F0 values

**vowel**	F1 Hz	F2 Hz	F1 Bark	F2 Bark	prom.	non prom.	prom.	non prom.
					F0 mean	F0 mean	F0 max	F0 max
/$\wedge$/	884	1545	7.8	11.29	281	235	307	249
/$\alpha$/	1056	1247	8.85	9.9	–	–	–	–
/$\upepsilon$/	870	1970	7.71	12.91	283	239	307	251
/æ/	1124	1810	9.24	12.34	–	–	–	–
/i/	594	2304	5.7	13.96	280	237	306	251
/ɪ/	307	3007	3.1	15.7	–	–	–	–
/u/	334	1363	3.37	10.47	284	232	306	248
/ʊ/	627	1648	5.97	11.72	–	–	–	–

After the F1/F2 continua were created, F0 was resynthesized to signal prominence, based on the naturally elicited productions from the model speaker. With the goal of keeping F0 resynthesis controlled while also being naturalistic, we resynthesized F0 to match naturally produced contours for a given contrast. For each contrast, a representative less-prominent contour (analogous to a H* pitch accent in ToBI annotation; Beckman & Hirschberg, 1994) was selected, and a representative more-prominent contour (analogous to a L+H* pitch accent) was selected. These were each overlaid on the continuum using the PSOLA method, in Praat with both conditions thus created by resynthesis (Boersma, 2021; Moulines & Charpentier, 1990).

We selected different F0 contours for each contrast, which served to introduce some naturalistic variation in the stimuli across contrasts and allow for the possibility that the different contrasts were produced with slightly different F0 contours. For all contrasts, the prominent condition shows a later and higher F0 peak, and overall higher F0. The F0 contours for each contrast and prominence condition are shown Fig. 1B and given numerically in Table 1.

The final step in the process was duration resynthesis. There were five continuum steps for each duration continuum. The duration continua end points were set according to the speaker’s productions. For each continuum, the shortest vowel duration was that of the “no-emphasis” production of the intrinsically shorter vowel, and the longest vowel duration was that of the “very emphasized” production of the intrinsically longer vowel. For each vowel contrast and each F0 condition, the continuum was resynthesized into five steps using a Praat script (Winn, 2014). The ranges for the duration continua, which were based on the speaker’s own productions of each vowel, were the following: /ɪ/-/i/ = 110–270 ms; /$\upepsilon$/-/æ/ 80–250 ms; /$\mho$/-/u/ = 150–320 ms; /$\wedge$/-/$\alpha$/ = 60–250 ms. A single production of “I will say *x* now” was selected from the “emphasized” production as the carrier phrase, then each of the 280 target word stimuli was embedded into the position of “*x*”. For the purpose of limiting the number of trials in the experiment, we dropped formant steps 2 and 6, keeping the endpoints of the continuum and the innermost three steps. There were therefore 200 unique stimuli (four contrasts $\times$ five formant steps $\times$ two F0 conditions $\times$ five durations).

We then added multi-talker babble noise in order to create what will henceforth be referred to as the noise condition. The babble noise was created based on the speech production of four speakers, which was collected from another previous study (Zhang et al., 2023). Babble noise with four speakers was chosen to elicit a moderate level of informational masking effect, as Mattys et al. (2009) classified one-speaker babble as causing strong informational masking and eight-speaker babble as causing little informational masking. The content of the speech was the reading production of the short passage “The North Wind and the Sun,” which contains 126 words. We extracted the first 30 s of each of the four speakers’ speech and divided them into ten 3-s segments. Next, we randomly selected one of the ten 3-s pieces from each speaker and combined them to create a 4-speaker babble noise. This process resulted in ten distinct 4-speaker babble noises, with no segment reused. In the final step, we randomly divided the quiet stimuli into ten groups, paired each group with one of the ten babble noises randomly, and mixed each quiet stimulus in each group with the paired noise to generate noise stimuli. To eliminate potential *energetic* masking in the babble noise, we presented the babble noise and the target sentence dichotically to the listeners (as in Symons et al., 2023; Mattys et al., 2009). Dichotic stimuli were made through the “Combine to stereo” function in Praat (Boersma, 2021). The stimuli were randomized to determine whether the babble noise was presented to the left or right ear. Following Chen et al. (2023), the signal-to-noise ratio was set 0 dB. Both the quiet and noise stimuli were rescaled to 70 dB. This resulted in a total of 400 unique stimuli, 200 in each noise condition. In a previous version of the experiment, we mixed the noise and target audio into a single channel, effectively introducing possible energetic masking for the target. These results from the previous experiment are included in the online “one channel” supplement for interested readers.

#### Experiment 2

While Experiment 1 is focused on the relative weighting and impact of noise on the perception of duration and formant cues, Experiment 2 is concerned with the relation of contextual and vowel-internal prominence, and the impact of noise for each. We modified a subset of the stimuli in Experiment 1 to make the stimuli for Experiment 2. Following the results of Experiment 1, we opted to focus on formant cue perception, thus taking one single duration step for each contrast based on what step showed the most balanced categorization across the formant continuum (duration step 2 for /$\wedge$/-/$\alpha$/, duration step 3 for the remaining three contrasts). From here, we created two new contextual F0 conditions. For both, the duration of the pre-target vowel in the word “say” was lengthened, which was judged to sound most natural in both F0 conditions and make the pre-target F0 variation more salient. In Experiment 1 the duration of this vowel was 114 ms, in Experiment 2 the duration of the vowel was lengthened to be 200 ms. Following this, the F0 on the voiced segments of the pre-target materials was raised to create the “high context F0” condition, and lowered to create the “low context F0 condition”. The original F0 for the precursor had a mean value of 209 Hz. The low context F0 condition had a mean F0 value of 184 Hz. The high context condition had a mean F0 of 250 Hz. These two F0 conditions from Experiment 2 are shown overlaid on a stimulus waveform in Fig. 1C. The values for the conditions were determined by auditory assessment of the authors, as values that were perceptually distinct, natural for the speaker’s F0 range, and plausible intonational melodies. Each contextual F0 condition was crossed with all other conditions to create 80 unique stimuli (four contrasts $\times$ five formant steps $\times$ two target F0 conditions $\times$ two context F0 conditions). Each stimulus was repeated two times in the experiment for a total of 160 trials without babble noise. Babble noise was added in the same manner as for Experiment 1, resulting in a total of 320 trials in the experiment, 160 in each noise condition.

### Participants and procedure

In each of the two experiments described in this paper, we recruited 50 participants (none of whom took part in both experiments). Participants were recruited online using Prolific, provided informed consent to participate, and were paid for their time. All participants were self-reported monolingual speakers of American English with no hearing difficulties or deficits. Participants were instructed to take the experiment while using headphones and in a quiet location. Both experiments consisted of two blocks, one with noise, and one without. Block order was counter-balanced across participants such that 25 (in each experiment) received the no noise (henceforth “quiet”) block first, and 25 received the noise block first.

The stimuli in both experiments were presented via the platform Testable (Rezlescu et al., 2020). On a given trial, listeners were presented with a token from that continuum and given orthographic response options (for example, after hearing a token from the /i/ - /ɪ/ continuum, listeners saw “keyed” and “kid” as response options on the screen). The stimulus played simultaneously with the presentation of response options, though a key press response could not be registered until the time in the stimuli at which the full target word had played, in order to prevent responses before the target was heard. The side of the screen on which target words appeared varied (e.g., in some trials for the /i/ - /ɪ/ continuum, “keyed” was on the left, and in others it was on the right), and pair of words appeared an even number of times on each side of the screen across the experiment. Participants registered their categorization response via keypress, using the 1 and 4 keys on the computer keyboard.

Before the experiment began, there were two headphone checks. In the first, after providing basic demographic information, participants indicated that they were indeed wearing headphones (already mentioned prior to the launch of the experiment, as a requirement), and they supplied the headphone make and model. After this, they were presented with a test stimulus that presented two-channel audio with a babble noise sample in one channel and piano music in the other channel. They were told that they should proceed if and only if they heard piano music in one ear and voices in the other ear. They were asked to leave the study if not, ensuring that all those participants who participated experienced successful dichotic presentation of the noise stimuli.

As noted above, in Experiment 1, there were a total of 400 trials, with 200 per noise condition. Within each noise condition block, the 200 trials were composed of 5 formant steps $\times$ 5 duration steps $\times$ 2 prominence conditions $\times$ 4 vowel contrasts. In Experiment 2, there were a total of 320 trials, with 160 per noise block. Within each noise condition block, the 160 trials were composed of 5 formant steps $\times$ 2 contextual F0 conditions $\times$ 2 prominence conditions $\times$ 4 vowel contrasts $\times$ 2 presentations of each stimulus.

For each of these experiments, we excluded any participants who did not show sensitivity to the formant continua in the stimuli.^1^ We excluded five participants from Experiment 1 (from an original total of 55 participants) and six participants from Experiment 2 (from an original total of 56 participants).

### Data analysis

All data, the scripts used to analyze the data, and the pre-run statistical models can be found online, hosted on the OSF at https://osf.io/52khc/.

We analyzed listeners’ responses using mixed effects Bayesian logistic regression, implemented in *brms* (Bürkner, 2017). We subsequently used *emmeans* (Lenth, 2023) to compute marginal contrasts of interest, and examine interactions between fixed effects in the model. We additionally used the *estimate slopes* function from the *modelbased* package (Makowski et al., 2020) to examine interactions that involve the continuous variables in the model (formant continuum step, and duration continuum step).^2^

When reporting results in what follows, we present two characterizations of evidence for an effect. Firstly, we present median posterior estimates from the model and relevant marginal contrasts, including 95% credible intervals (CrI) around these estimates, where the credible intervals indicate the lower and upper bounds of 95% the estimated posterior distribution. One conventional way of interpreting effects in Bayesian modeling is to examine the spread of the CrI, and critically, whether or not they include the value of zero. If yes, this suggests a non-trivial proportion of estimates are either at or near zero (no estimated effect), or showing substantial variability in the estimated directionality of an effect. In both cases, the estimate is taken to be unreliable as evidence for an effect. Conversely, CrI which exclude the value of zero, suggest a non-zero effect and a consistently estimated directionality, in which case an effect is taken to be “credible”. We additionally report the “probability of direction” for an estimated effect, computed using *bayestestR* (Makowski et al., 2019a, b), which is another way of numerically characterizing an estimated posterior distribution. This measure gives the percentage of an estimated distribution that sits on one side of the value of zero, ranging between 50 and 100. A distribution centered precisely on zero (no effect) would have a pd of 50, while one with all-negative or all-positives estimates would have a pd of 100. When 95% CrI exclude the value of zero pd = 97.5, though estimates approaching this threshold can also be interpreted in the Bayesian framework as graded evidence for effect existence, i.e., indexing the probability that an effect exists with a particular directionality (pd is considered an index of effect existence, as compared to other indices like Bayes Factors). For example, an estimate with a pd of 97 will have 95% CrI that narrowly include zero, nevertheless can be interpreted as indexing a 97% probability that the effect exists in that directionality. Interpretation of pd in this way has the benefit of seeing the estimates as continuous-valued strength of evidence without putting too much weight on arbitrary CrI (95%) or arbitrary pd thresholds (97.5). Given all of this, a note about how we interpret these effects is warranted. In the present study, we focus on effects which are “credible” with 95% CrI excluding zero, but also on effects with pds approaching this threshold, limiting ourselves to pds equal to or in excess of 95 (i.e., 95% chance an effect exists with a particular directionally). This value of 95 is arbitrary as well, but has the benefit of using pd as a metric that complements the binary threshold assessment of credibility, while also limiting ourselves to cases where there is a fairly strong degree of evidence for an effect (95% chance of effect existence or greater). In describing the results, we qualify effects such as these, where 95 < pd < 97.5 as being supported by “weaker evidence”. We do not consider pd values below 95 as providing support for an effect, though here again we must say this is an arbitrary threshold and the reader is welcome to interpret lower pd values directly as the probability of an effect’s existence in a particular direction. Here, too, it is important to note that pd values for complex interactions (e.g., three-way or four-way) can serve as evidence of the interaction’s existence. These interactions can be further explored by visualizing the relevant conditions or by computing marginal effects and slopes, as described above.

Here we detail the specific predictors that were present in the models for both Experiment 1 and 2. In both models, the dependent variable was listeners’ categorization response for each pair of vowels. To standardize responses we mapped the higher vowel in the pair (in canonical vowel space descriptions) to 1 and the lower vowel in the pair to 0 as with the individual participant analysis (/i,u,$\upepsilon$,$\wedge$/ mapped to 1 and (/ɪ,$\mho$,æ,$\alpha$/ mapped to 0). In both experiments, the formant continuum was centered and scaled, using the scale() function in base R, with numerically lower steps corresponding to the higher vowel endpoint for each contrast. In Experiment 1, the duration continuum was also centered and scaled, with higher numerical values corresponding to longer duration. Given this coding scheme, we expect that increases along the formant continuum will be associated with decreases in the log-odds of a higher vowel response (/i,u,$\upepsilon$,$\wedge$/). For duration, as found in Steffman and Zhang (2023), there is a predicted asymmetry based on vowel height (a predictor in the models). For high vowels, longer duration is associated with /i,u/, the two higher vowels in each pair. For non-high vowels, longer duration is associated with /æ,$\alpha$/ the two lower vowels in each pair. Therefore, longer duration should increase “higher vowel” responses for high vowels and should decrease “higher vowel” responses for non-high vowels. The remaining variables in the models were all two-level categorical variables, each of these was sum coded. For vowel height, high vowels were mapped to 0.5, and non-high vowels were mapped to -0.5. For the prominence variable, non-prominent was mapped to 0.5 and prominent was mapped to -0.5. For the noise manipulation, noise was mapped to 0.5, and quiet was mapped to -0.5. In Experiment 2, the introduction of the contextual F0 manipulation was coded with high contextual F0 mapped to 0.5, and low contextual F0 mapped to -0.5. Random effects in the model consisted of random intercepts for participant, with all fixed effects and interactions between fixed effects as by-participant random slopes. A random intercept was also included for vowel contrast.

Here an important terminological note should be reiterated: we use the term “high vowel” to refer to the two contrasts which contain only high vowels: /i/-/ɪ/ and /u/-/$\mho$/, whereas, as described above “higher vowel response” refers to the higher vowel *within each contrast*: /i,u,$\upepsilon$,$\wedge$/.

### Evaluating cue weighting and enhancement

Given that one of our key interests is in how both prosodic prominence and noise impact the use of formant and durational cues, here we describe how we operationalized cue weight in the models we present. We considered that higher cue weight for, e.g., vowel formants, would be reflected in a steeper categorization function along the continuum. This can effectively be computed by estimating the slope of the formant continuum (as a variable) at various levels of other variables, and can be indicated statistically in the model via interactions involving the formant continuum and another variable. For example, a two-way interaction between formant continuum and prominence would suggest that the formant continuum is categorized differently in each prominence condition; this was found in Steffman and Zhang (2023). Further examining the slope of the formant continuum at each prominence condition can reveal how they differ. A larger absolute value for the slope would thus indicate a steeper categorization function, or an effective enhancement or up-weighting of formant cues. As noted above, based on the variable coding scheme, the effect estimate is generally expected to be negative (i.e., decreasing log-odds of a higher-vowel response with numerical increases along the formant continuum). Steffman and Zhang (2023) found that there were higher absolute value slopes estimated under prominence, which we thus predict to observe here. The noise effect might also generally be expected to lessen the sharpness of the categorization function (smaller absolute slope estimates), as detailed above. The same prediction can be carried over to the duration continuum, where the presence, or absence of interactions between this continuum and the prominence and noise conditions can index how they modulate duration cue usage.

**Fig. 2:**
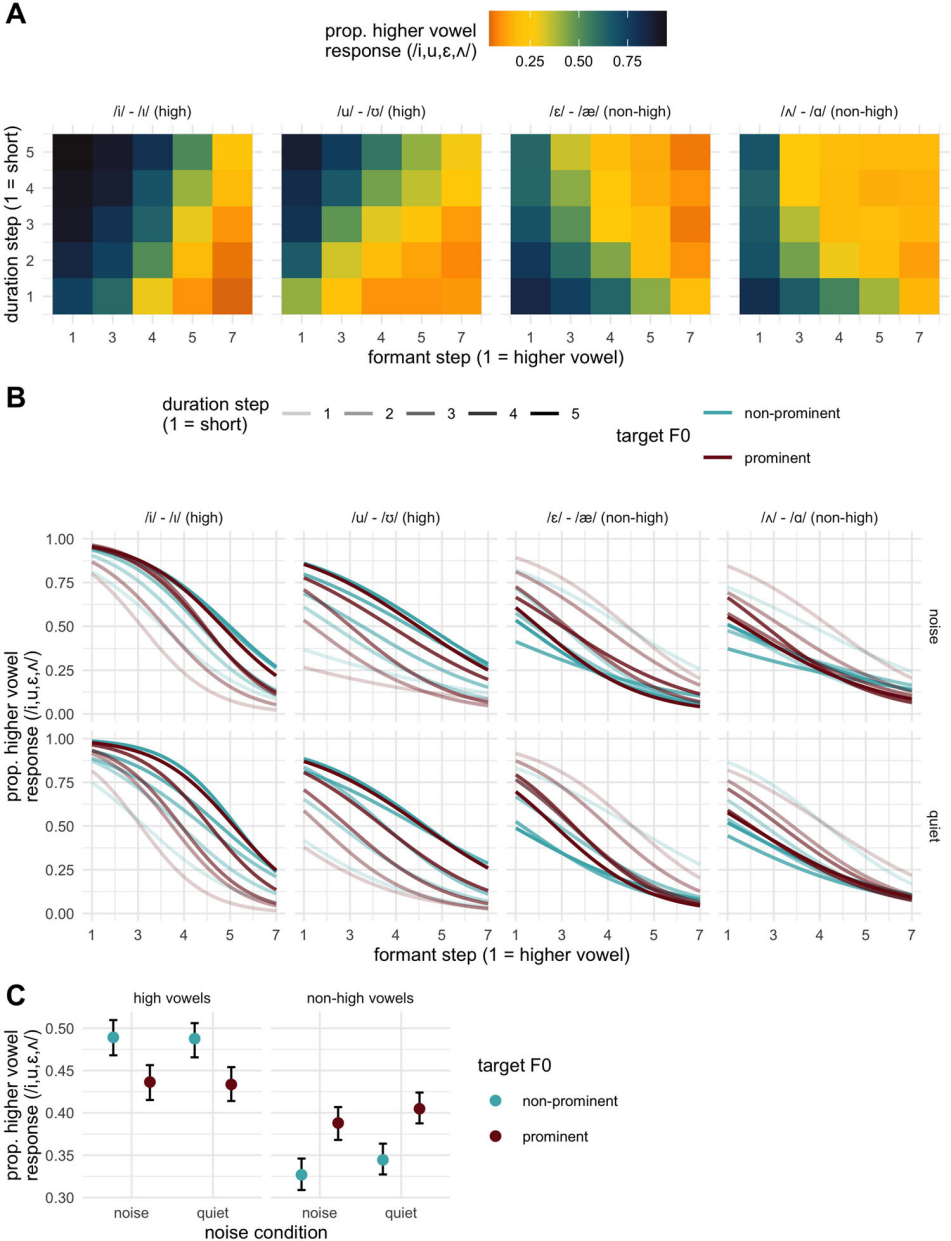
Empirical results from Experiment 1. **A** The proportion of a higher vowel response given to each step of the 5 $\times$ 5 duration and formant continua. **B** Higher vowel responses as a function of the formant continuum (*x*-axis), with line transparency indicating duration, line color indicating prominence, and each row indicating noise condition. Lines are psychometric curves fit to the data. **C** Higher vowel responses as a function of noise and prominence, and collapsed across both continua

## Experiment 1 Results

In presenting the results from Experiment 1, we first briefly outline the effects of formants, duration, and vowel height. We then turn to a discussion of the prominence and noise manipulations and their interactions with the other stimulus dimensions. We provide estimates, CrI, and pd for effects, both extracted from the model summary itself and computed with *emmeans* and *bayestestR*. We present just the probability of direction metric for estimates of interactions, as the actual estimate values are not easily interpretable for interactions given the coding scheme in the model structure.

The effects of formants, duration, and vowel height essentially replicated Steffman and Zhang (2023). There was an expected effect of formants ($\hat{\beta }$ = -1.81, 95% CrI = [-2.06, -1.56]; pd = 100), which also interacted with vowel height (pd = 98). The interaction evidenced a larger effect of formants for the high vowel contrasts ($\hat{\beta }$ = -1.91, 95% CrI = [-2.18, -1.64]; pd = 100) as compared to non-high vowels ($\hat{\beta }$ = -1.70, 95% CrI = [-1.96, -1.45]; pd = 100). This can be seen in the empirical data in Fig. 2, where high vowel contrasts show sharper changes in categorization in panel A (coloration in the figure) from left to right, and steeper slopes in panel B.

There was not a main effect of duration (pd = 73). According to our coding scheme for the dependent variable in the regression model, the two high vowel pairs (/i-ɪ/ and /u-$\mho$/) are expected to show reversed directionality compared to the two non-high pairs (/$\upepsilon$-ae/, /$\wedge$-d/) for duration as noted above. Since no clear predictions were made regarding the effect size, we did not have a priori expectations for an averaged positive or negative main effect of duration (given the sum coding of the vowel height variable). Accordingly, we examine the role of duration through the interaction effect of duration and vowel height. There was the predicted asymmetrical effect of duration across heights, reflected in a credible height by duration interaction (pd = 100). Non-high vowels showed a negative estimate ($\hat{\beta }$ = -0.88, 95% CrI = [-1.09, -0.66]; pd = 100), indicating that longer duration (numerical increases in the duration step variable) correspond to *decreases* in the log odds of a higher vowel response. This is in line with intrinsic vowel duration, where longer duration is expected to favor perception of /$\alpha$/ and /æ/. The opposite directionality for high vowels ($\hat{\beta }$ = 0.97, 95% CrI = [0.76, 1.17]; pd = 100) can also be interpreted as an intrinsic duration effect, where longer duration favors perception of the intrinsically longer /i/ and /u/. This asymmetry is clearly seen in Fig. 2A, where the two high vowel contrasts show a different pattern than the two non-high vowel contrasts, observing the changes associated with increasing the duration step along the *y*-axis, and can also be seen across the line opacity in Fig. 2B.

Moving onto the prominence effect, the critical prediction of a prominence by vowel height interaction was borne out (pd = 100), leading us to examine the prominence effect for each vowel height category. For high vowels there was a credible prominence effect ($\hat{\beta }$ = -0.52, 95% CrI = [-0.34, -0.71]; pd = 100), where prominence decreased higher vowel responses.^3^ For non-high vowels, prominent vowels showed increased higher vowel responses: ($\hat{\beta }$ = 0.36, 95% CrI = [0.53, 0.18]; pd = 100).^4^ Both of these effects are commensurate with perceptual peripheralization in the vowel space, replicating Steffman and Zhang (2023): listeners expect more extreme formant values under prominence and re-calibrate vowel perception accordingly. The prominence effects can be seen clearly in panel C of Fig. 2 on the overall rate of higher vowel responses for both high and non-high vowels. It can be noted that the prominence effect is robust in both noise conditions; the presence of noise did not modulate this prominence effect (cf. Bosker et al., 2017). This was concluded based on the fact that there was not strong evidence for a three-way interaction between prominence, vowel height, and noise (pd = 84), nor between prominence and noise (pd = 56).

Before moving on to a discussion of the noise and prominence effects on formant and duration perception, one other credible interaction was of note. This was the interaction between the two continuous variables, formant step and duration step ($\hat{\beta }$ = -0.13, 95% CrI = [-0.21, -0.07]; pd = 100). Given the directionality of the estimate, we can interpret this interaction as indicating a numerical increase in duration results in a more negative (i.e., larger) effect for changes in the formant step variable.^5^ This was confirmed by estimating formant slopes at shorter durations (scaled duration = -1; $\hat{\beta }$ = -1.67, 95% CrI = [-1.91, -1.43]), the mean duration (scaled duration = 0; $\hat{\beta }$ = -1.80, 95% CrI = [-2.06, -1.56]) and longer durations (scaled duration = 1; $\hat{\beta }$ = -1.94, 95% CrI = [-2.21, -1.68]), showing steeper (more negative) slopes are estimated as duration increases. This could be understood through the lens of longer duration increasing the perceptual prominence of the vowel, or simply allowing listeners more time to perceive formant information, in either case up-weighting formant cues. The absence of a three-way interaction between formant step, duration step, and vowel height (pd = 58) would instead suggest that the duration-based enhancement of formant cues is uniform across heights.

**Fig. 3:**
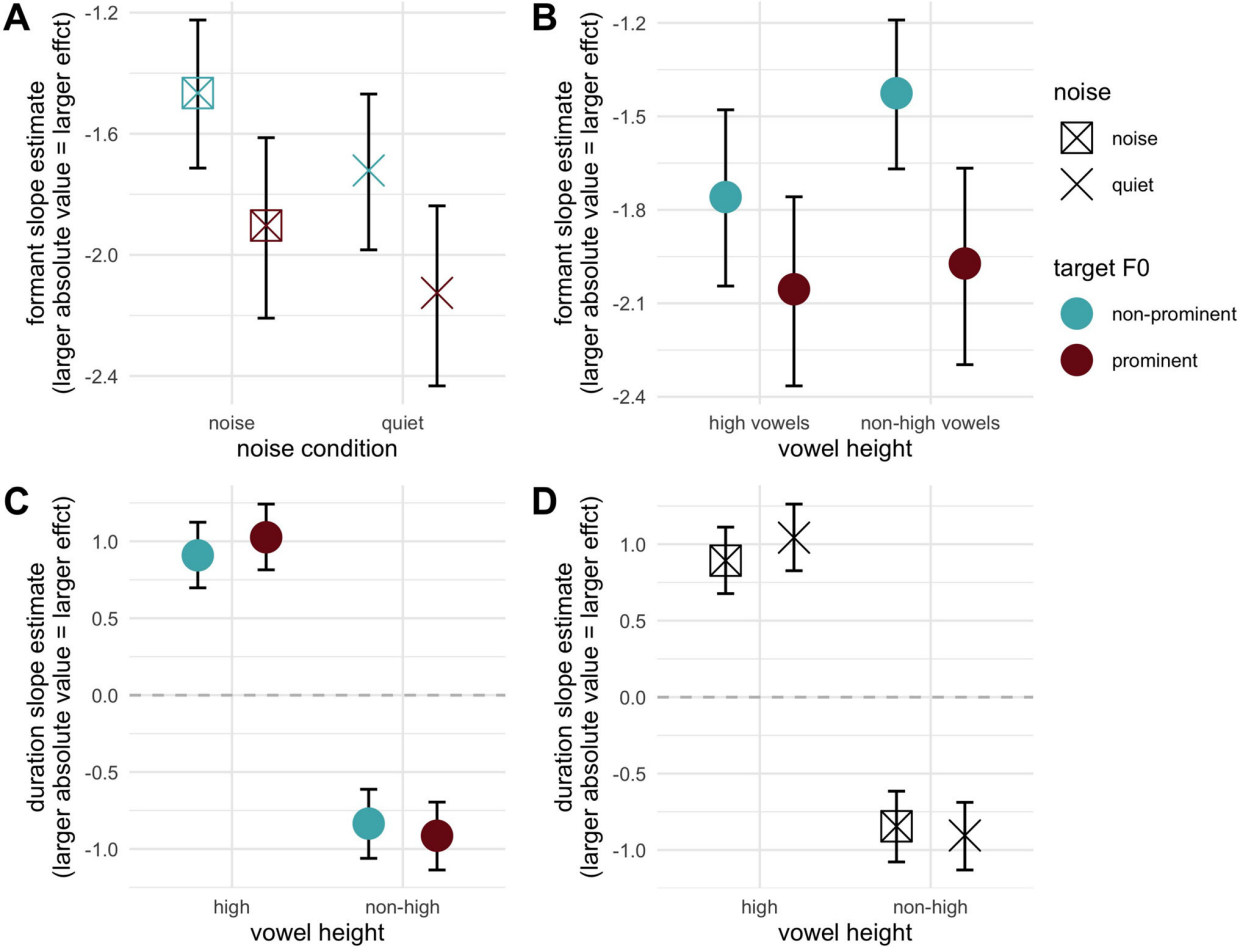
Slope estimates for the formant continuum (described in text). **A** Estimates split by noise (*x*-axis) and prominence (*color*, *indicated at right*), indicating the two-way interactions between noise and formant step, and prominence and formant step, which exert an additive (not interactive) influence on the slope estimate. **B** The estimates for prominence split by vowel height. **C**, **D** The estimates for duration slopes, both split by vowel height, and split prominence and noise, respectively

We can now consider how the prominence and noise manipulations interacted with formant and duration continua. The key focus of this section will be considering whether prominence-based enhancement of these cues exists across noise conditions, and how noise itself impacts cue usage. There were two key interactions of interest in this light. First an interaction between formant continuum and prominence was observed ($\hat{\beta }$ = 0.42, 95% CrI = [0.26, 0.59], pd = 100). The slope estimates for each prominence condition confirmed larger effects (steeper slope) in the prominent condition ($\hat{\beta }$ = -2.02, 95% CrI = [-2.30, -1.74]) as compared to the non-prominent condition ($\hat{\beta }$ = -1.59, 95% CrI = [-1.83, -1.36]). This broadly comports with the findings in Steffman and Zhang (2023), suggesting that prominence enhances the perception of formant cues. There was additionally a credible two-way interaction between the noise condition and formant continuum ($\hat{\beta }$ = 0.24, 95% CrI = [0.09, 0.39], pd = 100): a smaller/less-negative effect estimated in the noise condition). Slope estimates for each noise condition confirmed a larger effect (steeper slope) in the quiet condition ($\hat{\beta }$ = -1.92, 95% CrI = [-2.19, -1.67]) as compared to the noise condition ($\hat{\beta }$ = -1.68, 95% CrI = [-1.94, -1.44]). This suggests that, in a mirror image of the prominence effect, the dichotic presentation of babble noise in the stimuli reduces the sharpness of formant categorization. Both of these effects are visualized in Fig. 3A, which includes the estimates for noise and prominence conditions. Both two-way interactions are evident in noting the generally larger (more-negative) slope estimates between the quiet and noise conditions (the two rightmost points compared to two leftmost points) and in comparing the prominence conditions as indicated by the color of each shape. We can note too that these effects are additive, where a prominent vowel in the quiet condition has the steepest slopes, a non-prominent vowel in the noise condition has the least-steep slopes, and the other two conditions are intermediate. There was no evidence for a three-way interaction between formant continuum, noise, and prominence (pd = 61) further indicating that effects are additive and not interactive.

Because Steffman and Zhang (2023) found that prominence-based enhancement of formant perception was asymmetrical across vowel heights, we expected that we may observe it here too. The critical three-way interaction testing this is that between vowel height, formants and prominence. Here we found what can be characterized as weak evidence for the interaction, with credible intervals narrowly including the value of 0 ($\hat{\beta }$ = -0.25, 95% CrI = [-0.55, 0.03], pd = 96). To explore the interaction we visualized formant slope estimates in 3B, where it can be seen that there is a larger prominence effect on formant slopes for non-high vowels, whereas high vowels are less impacted, they show the same overall prominence enhancement effect. In comparison, the interaction between noise and formant continuum was not further mediated by vowel height (pd = 81).

Having now considered the effects of prominence and noise on the formant slope estimates, we consider if we can observe analogous evidence for duration, an evident additional cue to these contrasts, as shown in Fig. 2. Here, importantly, evidence for changes in duration slope will also interact with vowel height because the duration effect is height-dependent. Evidence for enhancement (for example) of duration under prominence would therefore be larger-valued positive estimates for high vowels, and larger-absolute-valued (more negative) negative estimates for non-high vowels, reflected in a three-way interaction therefore in the model. There was indeed credible evidence for this three-way interaction in the model, with CrI narrowly excluding zero ($\hat{\beta }$ = -0.20, 95% CrI = [-0.38, -0.00], pd = 98). To understand the interaction, the estimates for duration were visualized in Fig. 3C, showing that, as would be predicted from the enhancement lens, slope estimates are higher for high vowels, and more negative for non-high vowels under prominence. The estimates for the analogous interaction containing the noise variable were heavily skewed away from zero, with 95% CrI nevertheless narrowly including zero ($\hat{\beta }$ = -0.21, 95% CrI = [-0.42, 0.01], pd = 97). Whereas this evidence is not (quite) as robust as the prominence-containing interaction, it can still be taken to suggest that there is some asymmetry in duration slopes across height and noise variables. This is visualized in Fig. 3D, plotting the duration slope estimates as a function of these other two variables. These estimates show that the quiet condition shows larger absolute slope values for both high and non-high vowels, as compared to the noise condition. In other words, the quiet condition effectively mirrors the prominent condition in up-weighting duration as a cue.

In summary, the results of Experiment 1 show that prominence and noise manipulations engender opposite effects on the perception of formant cues. Moreover, their effects seem to be additive as suggested by both two-way interactions between noise and formants, and prominence and formants. In other words, the observed detrimental effect of noise in formant perception can effectively be mitigated by prosodic prominence, cued here by raised F0. The patterns for duration were notable in several regards. First, duration was clearly used as a cue to the contrasts by listeners, showing robust effects for which the directionality was contingent on vowel height. At the same time, duration was observed to play an enhancement role in the two-way interaction with formants: longer durations corresponded to steeper formant slopes. This suggests a clear duality for duration as cue: listeners consider it both as a “direct” cue to these vowel contrasts, and modulate their formant perception on the basis of duration, where longer durations are beneficial for formant perception. Finally, duration as a direct cue also appears to be influenced by F0-based prominence and by the presence of noise, although the evidence for noise-based modulation is weaker. In both cases, however, duration is given greater weight as a cue for prominent vowels (compared to non-prominent vowels) and in quiet conditions (compared to noisy environments).

## Experiment 2 Results

In reporting the results of Experiment 2, we first look at the results overall to see how effects which are shared with Experiment 1 translate to Experiment 2, and how the contextual F0 variable may have interacted with these effects. Then we specifically examine the interactions between variables and the formant continuum, testing if the same evidence for the modulation of formant cue usage obtains in Experiment 2.

**Fig. 4:**
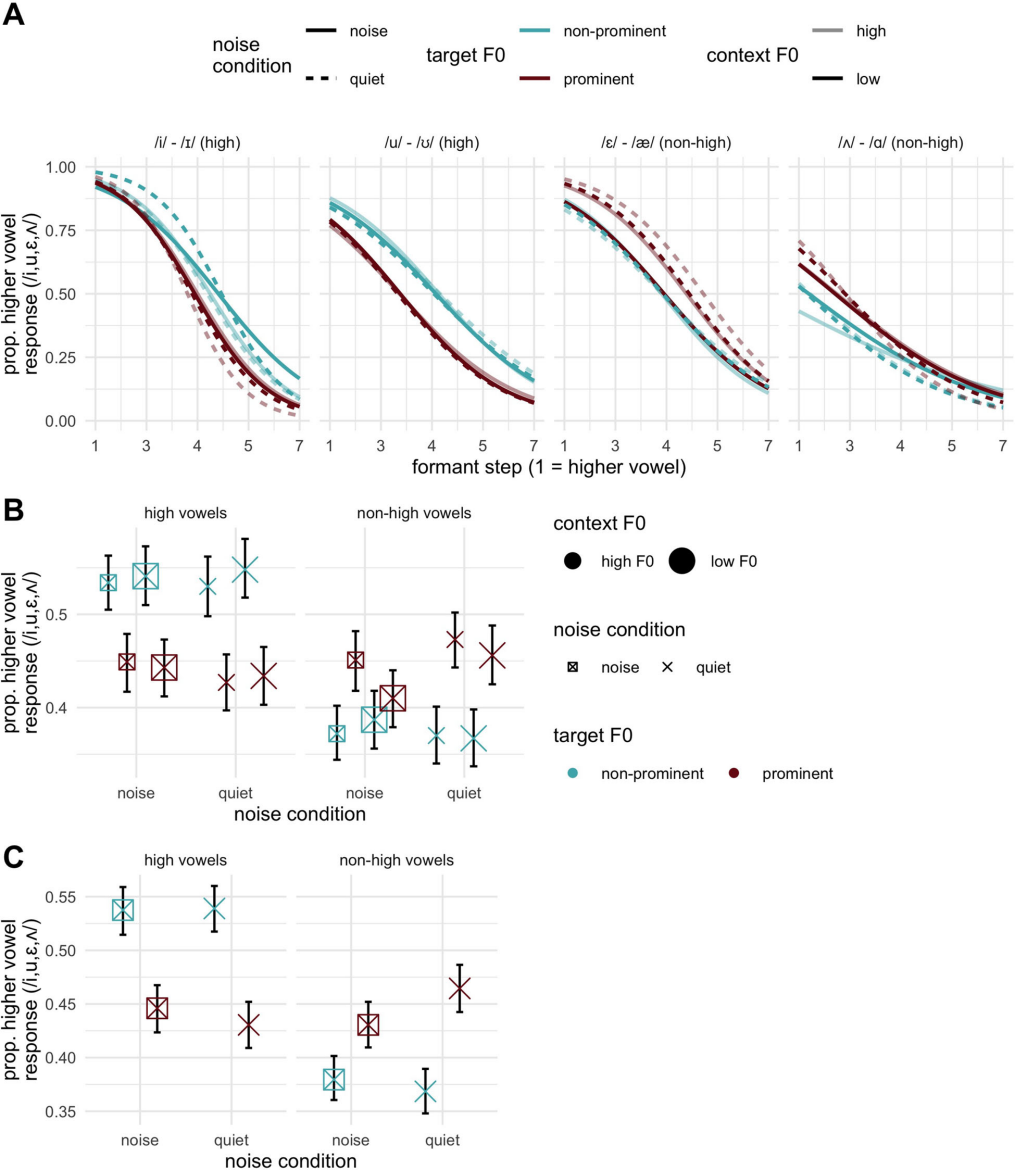
Empirical results from Experiment 2. **A** Higher vowel responses as a function of the formant continuum (*x*-axis), with line transparency indicating contextual F0, line color indicating prominence, and line type indicating noise condition. Lines are psychometric curves fit to the data. **B** Plots of higher vowel responses as a function of contextual F0, noise and prominence, collapsed across the formant continuum. **C** The same as panel B except that it pools across the contextual F0 variable to highlight the observed three-way interaction between prominence, vowel height, and noise

The effect of vowel formants was credible as expected ($\hat{\beta }$ = -1.99, 95% CrI = [-2.27, -1.72], pd = 100), and, as with Experiment 1, interacted with vowel height (pd = 100). Estimating the slopes for each height showed effects which comported with Experiment 1, whereby high vowels showed a larger effect ($\hat{\beta }$ = -2.27, 95% CrI = [-2.59, -1.96]) as compared to non-high vowels ($\hat{\beta }$ = -1.72, 95% CrI = [-2.01, -1.44]). This asymmetry can be seen in Fig. 4A, where overall categorization functions tend to be steeper for high vowels.

There was a main effect of prominence ($\hat{\beta }$ = 0.15, 95% CrI = [0.01, 0.31]) which further interacted with vowel height (pd = 100).^6^ Testing the prominence effect by vowel height further aligned with Experiment 1, wherein a height-dependent directionality for the effect reflected perceptual adjustment for prominence strengthening and peripheralization in the vowel space (high vowels: $\hat{\beta }$ = -0.83, 95% CrI = [-1.06, -0.59], pd = 100; non-high vowels: $\hat{\beta }$ = 0.52, 95% CrI = [0.29, 0.74], pd = 100).

**Fig. 5:**
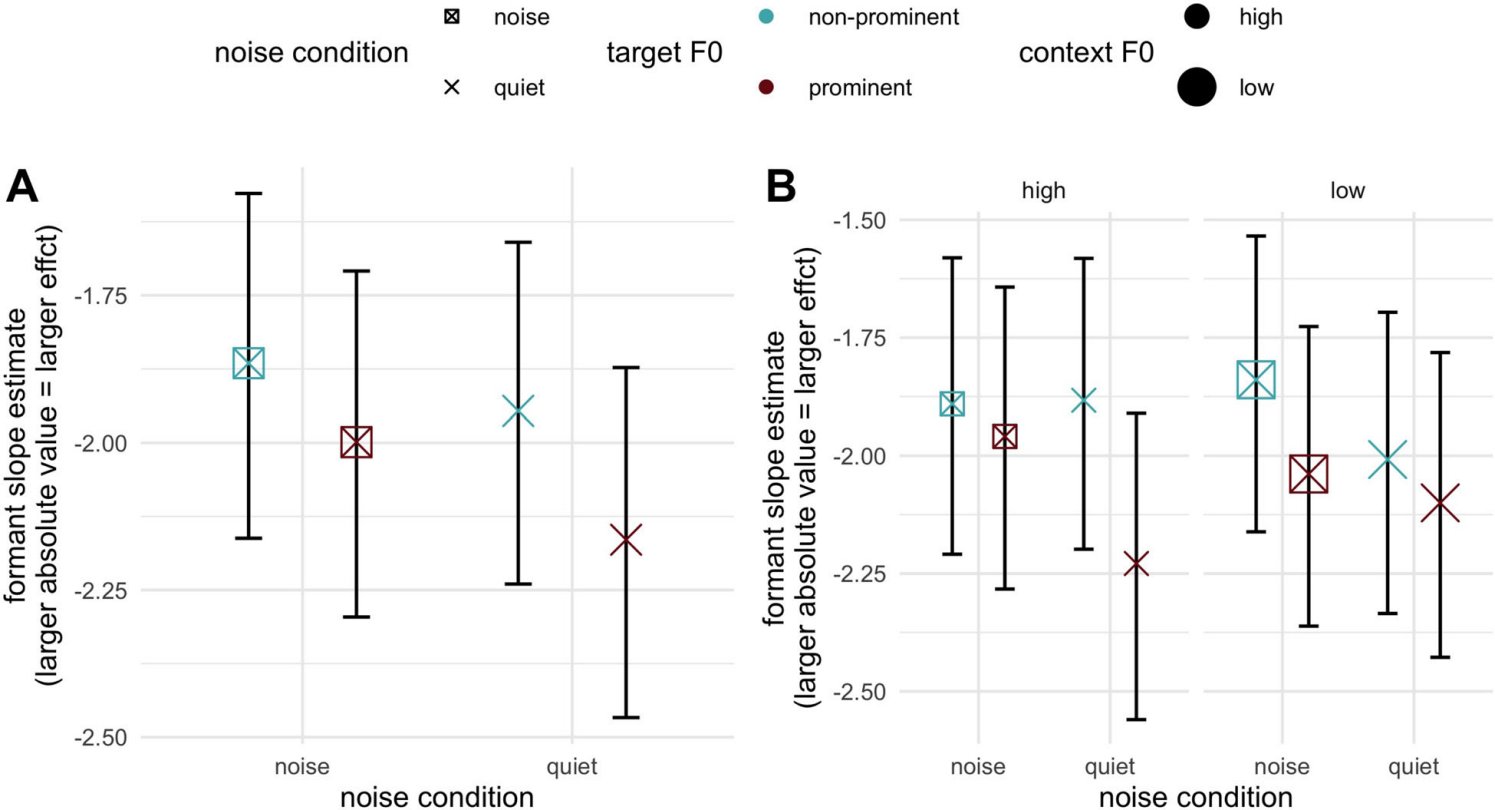
Slope estimates for the formant continuum in Experiment 2. The legend for all panels is shown at right. **A** Estimates split by noise (point shape) and prominence (point coloration, *indicated at right*), shows the two-way interactions between noise and formant step, and prominence and formant step, which exert an additive influence on the slope estimate. **B** The estimates further split by context, indicated by point size with high context F0 in the left sub-panel and low context F0 in the right sub-panel. *Error bars* indicate 95% credible intervals

There was additionally clear evidence for an interaction between noise, vowel height, and prominence ($\hat{\beta }$ = -0.47, 95% CrI = [-0.84, -0.10], pd = 99). The relevant data for this interaction is shown in Fig. 4C, where we can see the interaction can be understood in terms of the fact that the noise condition results in a smaller prominence effect for non-high vowels in particular, though it can also be noted the prominence effect does get slightly smaller in the noise condition for high vowels as well. While we did not predict this interaction, we think it may be explained as a decrement in listener’s ability to process prominence strengthening patterns when noise is present, a point returned to in the discussion. There was also weaker evidence for a context-prominence two-way interaction ($\hat{\beta }$ = -0.16, 95% CrI = [-0.35, 0.03], pd = 96), which did not interact further with vowel height (pd = 80). This reflected a larger effect of prominence in the low context condition ($\hat{\beta }$ = -0.23, 95% CrI = [-0.41, -0.06]) as compared to the high context condition ($\hat{\beta }$ = -0.07, 95% CrI = [-0.25, 0.10]). If we consider how this relates to vowel heights, a larger negative effect estimated across vowel heights would entail a larger negative effect for high vowels, and a smaller positive effect for non-high vowels, in the low context condition. Low context F0 corresponds to a very slightly larger prominence effect for high vowels, but a clearly smaller prominence effect for non-high vowels. The supplementary figure exp2_emp.2chan.context.prominence.jpg contained on the OSF displays the proportion of higher vowel responses by height, context F0 and prominence, showing this pattern, which can also be gleaned from Fig. 4B, visually averaging over the noise conditions. This point is returned to in the discussion.

We now consider the question of how formant slope estimates are modulated by prominence, noise, and contextual F0. As in Experiment 1, a credible formant by prominence interaction was observed ($\hat{\beta }$ = 0.18, 95% CrI = [0.05, 0.31]), pd = 100, with the same directionality as in Experiment 1. The two-way interaction between noise condition and formant step was not as strong, with CrI narrowly including zero and a pd value of 97 ($\hat{\beta }$ = 0.12, 95% CrI = [-0.01, 0.25]), i.e., we can be 97% sure that the estimates have this directionality for the interaction, which is also commensurate with Experiment 1. Both of these effects are shown in Fig. 5A, where formant slope estimates are plotted by both of these variables, showing, as with Experiment 1, slope estimates are larger (more negative) under prominence and in the quiet condition.

There was limited evidence for an effect of contextual F0. The two-way interaction between formant step and context F0 was not credible nor approaching it (pd = 55). It was worth noting that weak evidence was found for an interaction between contextual F0 and vowel height ($\hat{\beta }$ = -0.15, 95% CrI = [-0.33, 0.03], pd = 95), suggesting a similar off-target (though smaller) effect as on-target manipulation (prominence). No higher-order interactions including context and formants were credible, with one exception. This is the four-way interaction between formants, context F0, prominence, and noise, for which the estimates were strongly skewed ($\hat{\beta }$ = -0.38, 95% CrI = [-0.85, 0.09], pd = 95) though still encompassing the value of zero and hence not strong evidence for the interaction. As an exploratory investigation of this four-way interaction, we plot formant slopes by context, noise, and prominence in Fig. 5B, effectively splitting estimates in Fig. 5A by context. The following asymmetry can be noted: high context F0 (leftmost four points in panel B) shows fairly similar slope estimates for all conditions except for a prominent vowel in quiet, which has a larger (negative) slope value: in other words, only this optimal condition of prominence and no noise results in steeper slopes when contextual F0 is high. Conversely, for low context F0, the prominence and noise effects appear more additive, but still with all conditions but one having similar slope values. The odd-condition-out this time is the non-prominent condition in noise, the least optimal condition for formant slopes overall. In other words, conditions which are less optimal in *one way*, being either non-prominent in quiet, or prominent in noise have their slopes boosted by a preceding low F0 context, which we predicted should be beneficial in general.

What we can say definitively from the analysis of Experiment 2 is the core effects from Experiment 1 were replicated, including the interaction between vowel height and prominence, vowel formants and prominence, and to a slightly lesser extent (pd = 97) vowel formants and noise. The contextual F0 manipulation was very limited in its influence, showing weaker evidence for an interaction with vowel height (pd =95), an interaction with prominence (pd = 96) and participation in the higher-order interaction described in the preceding paragraph (pd = 95). From this, we conclude that on-target prominence is clearly more relevant than context both in terms of how it modulates vowel perception (in line with peripheralization of prominent vowels in the vowel space) and in the way it enhances processing of formant cues.

## Discussion and conclusions

The present study examined the attentional effects of prosodic prominence and informational masking on the perception of high and non-high vowel contrasts from a cue weighting perspective. The first experiment focused on their effect on listeners’ relative reliance on vowel formants (F1 and F2) and vowel durations. The second experiment further explored the effect of prosodic prominence through a combination of on-target F0 manipulation and contextual F0 manipulation. We summarize our results as follows.

First, we largely replicated core findings in Steffman and Zhang (2023). Experiment 1 showed again that the reliance of both formants and duration were vowel height-dependent, with a larger effect of formants for high vowels and a height-dependent directionality of duration effects, which is consistent with intrinsic vowel duration differences. While the duration effect was not examined in Experiment 2, the height-dependent formant effect was observed there as well. Both experiments further replicated that the on-target prominence effect was height-dependent (for high vowels, prominence decreased higher vowel responses and for non-high vowels, prominence increased higher vowel responses), which is commensurate with the perceptual adjustment for peripheralization in the vowel space, as proposed in Steffman and Zhang (2023). Additionally, both experiments replicated the enhanced perception of formant cues via F0-based prominence, with steeper formant slopes observed under prominence. Experiment 1 also replicated similar formant cue enhancement by longer duration, and further showed that vowel height interacted with this enhancement, highlighting the dual roles of duration as both a ’direct’ cue to vowel contrasts and as a mechanism that enhances formant perception. However, unlike Steffman and Zhang (2023), who found no evidence that prominence influences the weighting of duration as a direct cue to vowels, our Experiment 1 demonstrated that prominence up-weighted duration in categorization.

Second, the dichotic babble noise manipulation generally showed an opposite effect of prominence, and their effects exhibited an additive pattern. Both experiments showed that noise diminished perception of formant cues in categorization: the presence of noise reduced formant slope estimates (Fig. 3A), contrasting with the impact of prominence (Fig. 3B). Experiment 1 showed that noise diminished duration as a cue too through down-weighting of duration in the noise condition (Fig. 3D), also mirroring prominence’s up-weighting effect on duration (Fig. 3C). In both experiments, effects from noise and prominence were additive, as suggested by their non-credible interactions and their aggregate influence on formant slopes: formant slope, which indexes the efficiency of vowel perception, is steepest in the prominent and quiet condition, least steep in the non-prominent noise condition, and intermediate for the other two conditions (Figs. 3A and 5A). Notably, Experiment 1 showed that prominence and noise did not interact with duration in two-way interactions, however, they exhibited three-way interactions with duration and vowel height. The results suggested that the height-dependent duration cue to the vowel contrasts can be modified by attention modulations. However, the two types of attention modulations also show distinct properties. Unlike prominence, dichotic babble noise and its interaction with formants were not further modified by vowel height, revealing that the distraction induced by noise is symmetrical across both vowel heights.

Third, the contextual F0 manipulation in Experiment 2 elicited a more limited prominence effect, clearly weaker than the on-target F0 manipulation. The results of Experiment 2 did not show any main effect of contextual F0 manipulation, however, evidence was found that it did interact with prominence to some degree. First, overall, lower contextual F0 increased the prominence effect (although supported by a borderline pd) as compared to higher contextual F0. Second, a four-way interaction (Fig. 5B) revealed that higher contextual F0 increased the formant slope for prominent vowels in quiet, and that lower contextual F0 decreases the formant slope for non-prominent vowels in noise. These patterns loosely align with our prediction that off-target F0 variations can manipulate prosodic prominence.

The following discussion focuses on results in the second and third points.

### Attention modulation by prominence and noise

Results in this study showed oppositional effects of prosodic prominence and babble noise on vowel categorization, quantified via their respective influences in modulation of listeners’ categorization of vowel formants and duration. Specifically, prominence increases the use of both cues in vowel categorization, whereas babble noise decreases reliance on them. This aligns with the understanding of prosodic prominence, signaled by both F0 and duration as orienting listeners’ attention to the target (De Jong, 2004; Steffman & Zhang, 2023), thereby benefiting the perception of cues, and the expectation of babble noise as distracting listeners’ attention from the target (Mattys et al., 2013b), thereby impairing cue perception. In that sense, the data patterns clearly support De Jong (2004) in the conception of prominence as an attention-orienting mechanism.

The oppositional modulation on attention, i.e. the effects of prominence and babble noise-was evident to be additive in vowel categorization. The formant slope, which indexes the efficiency of the vowel perception, is steepest in the prominent and quiet condition, least steep in the non-prominent noise condition, and intermediate for the other two conditions. This finding suggests that enhanced prosodic prominence could compensate for the detrimental effects of babble noise by facilitating a more efficient allocation of attentional resources during vowel categorization. Conversely, the distraction of informational masking in formant perception can also be mitigated by prosodic prominence. As a result, prominent vowels in quiet conditions provide the most optimal environment for vowel categorization, whereas non-prominent vowels in noisy conditions represent the least optimal scenario.

Although the two types of attention modulation show many symmetrical effects, one discrepancy between the prominence and dichotic babble noise modulations was whether their effects were further modified by vowel height. Prominence effect as well as its interaction with formant cues, was contingent on vowel height, whereas the effect of noise and its interaction with formants were not. The influence of prominence on vowel categorization reflects perceptual compensation of vowel peripheralization, for which the movement was vowel height-dependent. In contrast, dichotic babble noise plays a role by broadly lowering listeners’ attentional resources when perceiving the vowels, operating at a cognitive level and thus being less sensitive to the phonetic properties of the vowel contrasts. Such differences suggest distinct underlying mechanisms of the two attentional modulations.

While this study focuses on informational masking, other studies have also examined energetic masking effects on cue usage in contrast categorization, providing a broader picture for speech perception in adverse conditions. DiNino (2024) investigated the spectral and durational contributions in /$\upepsilon$/-/æ/ categorization in both quiet and broadband speech-shaped noise (a type of energetic masking) conditions. They found down-weighting of formants and up-weighting of duration in their noise condition, whereas our results showed down-weighting of both formants (Fig. 3A) and duration (Fig. 3D) in noise. Although both types of noise exert detrimental effects on formant perception, listeners’ higher-level cognitive processing appears to reflect a different pattern. Under energetic masking, spectral cues become less useful but the informativeness of duration should be the same. As a result, we can hypothesize that listeners actively up-weight the secondary cue to aid vowel identification. In contrast, informational masking disturbs processing at a higher cognitive level, preventing listeners from retaining- or up-weighting any cue in the task. In other words, the results highlight the key distinction: energetic masking reduces the informativeness of spectral cues, whereas informational masking impairs listeners’ attentional resources, limiting their ability to utilize any cue effectively.

Although numerous studies (DiNino, 2024; Gordon et al., 1993; Kim, 2019) including the present one have found that noise modulates the weightings of both primary (formants) and secondary (duration) cues, other studies observed only the primary cue (Kong & Lee, 2018) or only the secondary cue (Symons et al., 2023) was impacted, in categorization experiments of stop contrasts in Korean and in English. Since these studies examined differing contrasts and features, the mixed results appear to reveal that the adjustment of cue reliance under attention manipulation is not only informativeness-dependent (i.e., whether a cue is primary or non-primary), but also contrast- and feature-dependent, and even noise-type dependent.

The adjustment of duration cue weighting under attention manipulations provides insight into the complex role duration plays in vowel perception. First, duration is used as a secondary cue assisting vowel contrast categorization, as evidenced by its height-contingent effects in Experiment 1 and in Steffman and Zhang (2023). Second, longer durations enhance listeners’ perception of formants, similar to the enhancement effect of F0-based prominence, possibly resulting from the fact that longer durations lend prominence. Notably, the height-dependent role of duration (the role in the First point) also appears to be modulated by both attention manipulations, as indicated by the three-way interactions among duration, vowel height and prominence or noise. However, the enhancement role (the role referenced in the Second point) does not exhibit such an interaction. These results suggest that attention modulations interact with duration’s cue-to-contrast role but not with its enhancement role, further delineating the distinct manners in which duration influences vowel perception.

### On-target vs. off-target prominence effects

Although prominence is a relative variable that can, in principle, be realized by manipulating context, results in the present study show that off-target prominence manipulation plays a much weaker role than on-target prominence manipulation. Unlike in Steffman (2021b, 2021a), we did not observe that off-target prominence directly induces compensatory effects as on-target prominence does. However, we did find evidence that off-target prominence (i.e., a lowered contextual F0) boosted the on-target prominence effect, both directly (in the two-way interaction with prominence) and indirectly (in a four-way interaction). In particular, the indirect boosting effect of context visualized in Fig. 5B shows that listening conditions which are less optimal in one way, being either non-prominent in quiet, or prominent in noise, have their slopes boosted by a preceding context F0, which we predicted should be beneficial in general. In this way of interpreting the four-way interaction, in a high F0 context, formant slopes are only really boosted in the optimal circumstances (prominent vowels in quiet) while in the low F0 context, they are boosted in all but the least-optimal (non-prominent in noise). This loosely aligns with our predicted enhancement of slopes in the low F0 context; however, given the limited robustness of the interaction, this exploratory interpretation should be treated with caution.

One potential reason for the difference from Steffman (2021a, 2021b) is the method of off-target prominence manipulation. Steffman (2021a, 2021b) manipulated the F0, duration, and amplitude of the context preceding a target vowel, whereas in the present study, only the contextual F0 is manipulated. This could explain why the off-target prominence in this study only plays a boosting role as an additive effect to the on-target prominence, which is a weaker one than observed in Steffman (2021a, 2021b). Future tests of this idea could vary the strength and type of contextual prominence cues to observe their relative contribution.

### Conclusion

The core result of this study is that prominence enhances listeners’ attention while dichotically-presented babble noise distracts listeners’ attention in vowel perception, as measured from a cue weighting perspective. Moreover, the effects of prominence and babble noise are additive, making prominent vowels in quiet the most optimal perception condition, and non-prominent vowels in noise the least optimal. Finally, although much weaker than the on-target prominence, off-target F0 manipulations also exerted an influence, primarily by amplifying the effects of on-target prominence and interacting with noise.

The findings of this study suggest several directions for future research. This study investigated informational masking (i.e., babble noise) only, the extent to which energetic masking, or other cognitive load manipulations, behave similarly to informational masking in vowel perception can be further studied, especially in, for example, energetic masking as in DiNino (2024) explored with multiple contrasts, or in interaction with informational masking. Second, more methods of contextual manipulation to elicit prominence (e.g., semantic focus and signal-independent discourse context as in Akker & Kutler, 2003; Bishop, 2012) may be explored in future studies to understand to what extent various other prominence cues mediate cue weighting in vowel perception.

## Data Availability

The data for both experiments is available on the OSF at: https://osf.io/52khc/.

## Appendix

**Table 2:** Full model summary for fixed effects estimates from Experiment 1

	Estimate	Est. Error	Lower 95% CrI	Upper 95% CrI	pd
Intercept	-0.61	0.50	-1.55	0.50	91
formants	-1.81	0.12	-2.06	-1.56	100
duration	0.05	0.08	-0.10	0.20	73
prom	0.08	0.05	-0.02	0.19	94
height	0.63	0.81	-1.15	2.15	81
noise	0.01	0.08	-0.15	0.17	55
formants:duration	-0.13	0.03	-0.21	-0.07	100
formants:prom	0.42	0.08	0.26	0.59	100
duration:prom	-0.02	0.05	-0.11	0.07	67
formants:height	-0.21	0.10	-0.41	-0.01	98
duration:height	1.84	0.15	1.56	2.14	100
prom:height	0.88	0.15	0.59	1.17	100
formants:noise	0.24	0.08	0.09	0.39	100
duration:noise	-0.04	0.05	-0.15	0.06	80
prom:noise	-0.01	0.08	-0.18	0.16	56
height:noise	0.10	0.15	-0.20	0.40	74
formants:duration:prom	-0.01	0.05	-0.12	0.09	61
formants:duration:height	-0.01	0.06	-0.13	0.10	58
formants:prom:height	-0.25	0.15	-0.55	0.03	96
duration:prom:height	-0.20	0.10	-0.38	-0.00	98
formants:duration:noise	-0.07	0.05	-0.17	0.03	91
formants:prom:noise	0.03	0.12	-0.18	0.27	61
duration:prom:noise	0.05	0.09	-0.12	0.22	73
formants:height:noise	-0.11	0.12	-0.36	0.13	81
duration:height:noise	-0.21	0.11	-0.42	0.01	97
prom:height:noise	-0.17	0.18	-0.53	0.18	84
formants:duration:prom:height	-0.16	0.11	-0.38	0.06	92
formants:duration:prom:noise	0.08	0.11	-0.12	0.30	79
formants:duration:height:noise	-0.06	0.10	-0.26	0.14	73
formants:prom:height:noise	-0.15	0.20	-0.55	0.25	77
duration:prom:height:noise	-0.24	0.17	-0.58	0.11	92
formants:duration:prom:height:noise	0.24	0.21	-0.17	0.65	87

**Table 3:** Full model summary for fixed effects estimates from Experiment 2

	Estimate	Est.Error	Lower 95% CrI	Upper 95% CrI	pd
Intercept	-0.29	0.59	-1.46	0.95	73
formants	-1.99	0.14	-2.27	-1.72	100
context	0.03	0.05	-0.06	0.12	73
prom	0.15	0.08	0.01	0.31	98
height	0.44	0.92	-1.5	2.19	71
noise	0.00	0.08	-0.16	0.16	51
formants:context	0.01	0.06	-0.11	0.12	55
formants:prom	0.18	0.06	0.05	0.31	100
context:prom	-0.16	0.1	-0.35	0.03	96
formants:height	-0.55	0.13	-0.81	-0.3	100
context:height	-0.15	0.09	-0.33	0.03	95
prom:height	1.35	0.18	1.00	1.69	100
formants:noise	0.12	0.06	-0.01	0.25	97
context:noise	0.06	0.10	-0.13	0.26	75
prom:noise	0.11	0.10	-0.08	0.30	87
height:noise	0.14	0.15	-0.15	0.43	83
formants:context:prom	0.06	0.14	-0.21	0.33	67
formants:context:height	-0.09	0.11	-0.32	0.13	79
formants:prom:height	-0.10	0.15	-0.4	0.18	76
context:prom:height	0.15	0.19	-0.21	0.52	80
formants:context:noise	0.02	0.12	-0.23	0.26	56
formants:prom:noise	-0.08	0.12	-0.33	0.16	75
context:prom:noise	-0.16	0.18	-0.52	0.19	82
formants:height:noise	-0.11	0.13	-0.37	0.13	82
context:height:noise	0.11	0.18	-0.25	0.46	72
prom:height:noise	-0.47	0.19	-0.84	-0.1	99
formants:context:prom:height	-0.23	0.25	-0.76	0.25	82
formants:context:prom:noise	-0.38	0.24	-0.85	0.09	95
formants:context:height:noise	-0.09	0.23	-0.54	0.37	65
formants:prom:height:noise	-0.04	0.24	-0.50	0.43	57
context:prom:height:noise	0.28	0.36	-0.44	0.98	78
formants:context:prom:height:noise	-0.62	0.47	-1.56	0.32	91

## Footnotes

1 This was accomplished by carrying out by-participant logistic regression analyses using the *broom* package in R (Robinson, 2014). These models predicted the log-odds of a response as a function of the vowel formant continuum (centered and scaled in the models), for each participant. We aggregated vowel responses from all four contrasts for this purpose, with the higher (in the vowel space) vowel in each contrast (/i,u,$\wedge$,$\upepsilon$/) being coded as 1. Given the way that the formant step variable was coded, an increase in the numerical steps along the continuum is predicted to decrease the log-odds of /i,u,$\wedge$,$\upepsilon$/ responses. We excluded participants who did not show a significant effect of formants in their perception of the aggregated contrasts, using the criterion of *p* < 0.05 to determine significance. A lack of an effect would suggest either that participants did not perceive the continuum endpoints as intended, or, that they were distracted, guessing at random, or otherwise not making a good-faith attempt at the study.

2 Models for both experiments were fit with a no U-turn sampler and the adapt delta parameter in the model set to the value of 0.99. In each of four Markov chains, 6000 samples were drawn from the posterior distribution over parameter values. Chains were run with a burn-in period of 1000 iterations, such that we retained the latter 5000 samples in each chain for inference.

3 Note that in this paper when inspecting the prominence effect by *emmeans*, we reported effect of the presence of prominence (instead of non-prominence) for easier interpretation (this simply flips the sign of the estimate but does not change value of the estimate). This reversal was achieved using the ‘revpairwise’ function in *emmeans*. This is the reverse of how the ‘prom’ term was coded in the models, where ‘non-prominent’ condition was coded as 0.5, and ‘prominent’ condition as -0.5.

4 There was some evidence for a main effect of prominence, with the estimated distribution was also clearly not centered on zero (pd = 94). This reflects the larger-magnitude effect seen for high vowels.

5 This broadly aligns with Steffman and Zhang (2023), who found that a similar duration-based enhancement effect for formant cues was observed, though in their case only for non-high high vowels.

6 As in Experiment 1. The positive sign of the main effect is such that the non-prominent target F0 condition results in increased higher vowel responses, which is the directionality observed for this effect for high vowels in Experiment 1.

## Code Availability

The code used to analyze both experiments is available on the OSF at: https://osf.io/52khc/.

## Declarations

### Code Availability

The code used to analyze both experiments is available on the OSF at: https://osf.io/52khc/.
